# Validation of Chemical Inactivation Protocols for *Henipavirus*-Infected Tissue Samples

**DOI:** 10.3390/v18010081

**Published:** 2026-01-07

**Authors:** Daniela Silva-Ayala, Anthony Griffiths

**Affiliations:** 1 National Emerging Infectious Diseases Laboratories, Boston University, Boston, MA 02218, USA; d.silvaayala@missouri.edu; 2 Department of Microbiology and Immunology, Bond Life Sciences Center, University of Missouri, Columbia, MO 65212, USA; 3 Laboratory for Infectious Disease Research, University of Missouri, Columbia, MO 65211, USA

**Keywords:** ABSL-4, virus inactivation, select agents, Nipah virus, negative sense RNA viruses

## Abstract

Biocontainment laboratories often have limited access to a range of instruments required for conducting standard assays on infected materials. Consequently, some of the protocols involving infected samples are conducted outside a biocontainment facility. To be compliant with regulatory requirements and minimize health and safety risks for scientific personnel, it is imperative to test procedures rigorously for safely removing infected samples from biocontainment areas. This study validated the chemical inactivation of Nipah virus (NiV), a representative member of the *Henipavirus* genus, in animal tissues and serum. Importantly, this work demonstrated successful NiV-spiking of non-human primate (NHP) tissues and their subsequent inactivation. This is important because NHP tissues contain unpredictable amounts of infectious virus. The primary objective was to establish standardized protocols that are compliant with regulations to permit safe retrieval of infected biological samples with high NiV infectious virus content from ABSL-4 laboratories for subsequent downstream processing under lower biocontainment conditions.

## 1. Introduction

Select Agents are biological agents that have been identified as having the capacity to pose significant risks to public health, safety, as well as animal and plant well-being, or the quality of animal and plant products, as indicated by the Select Agents and Toxins Program [1]. There are 68 Select Agents and Toxins that are overseen by the Federal Select Agent program (FSAP) [2].

Several viruses have been designated as Select Agents due to their ease of transmission, high infectivity, and that infection in humans frequently results in often-fatal diseases with limited or no available treatment options. Research conducted on these viruses may necessitate specialized facilities equipped with the highest levels of biosafety measures. Due to logistical constraints within a BSL-4 laboratory, research materials harboring infectious agents are often removed from containment areas for additional analyses. To maintain biosecurity and biosafety, the Select Agent program has strict requirements covering the processes surrounding the inactivation of samples to be removed from containment. Effective inactivation of viruses leads to the inability to replicate in a host. Importantly, the Select Agent program allows for the validation of inactivation procedures for only a single member of any given family of viruses to cover the entire family. Although some inactivation protocols are well established and even published, the Select Agent program requires that each inactivation protocol be appropriately validated at each facility [2].

Nipah virus (NiV) and Hendra virus (HeV) are bat-borne viruses considered category C (USDA-HHS overlap) Select Agents that can infect both humans and animals [1]. They belong to the Paramyxoviridae family, the same family of viruses as measles, mumps, and respiratory syncytial virus (RSV) [3,4,5]. Nipah virus was first identified in 1998 during an outbreak in Malaysia, and between 1998 and 2015, there were around 600 cases of NiV infection reported, according to the WHO. Since May 2023, the Southern Indian state of Kerala has been battling another deadly outbreak of the Nipah virus, the fourth since 2018. NiV is highly contagious, and there are no licensed medical countermeasures; work with authentic NiV must be performed in biosafety level 4 facilities (BSL-4) [6,7,8].

There have been many published inactivation protocols for enveloped RNA viruses when present in a variety of matrices (e.g., animal fluids, tissues, cell culture) [9,10,11,12,13,14,15,16,17,18]. Commonly used inactivation methods include heat, radiation, chemical treatments, like TRIzol reagent and 10% neutral buffered formalin. The choice of inactivation method is dictated by the downstream application. For NiV virus inactivation validation in tissues, the generation of samples with high virus content has been historically challenging. The literature reporting on the infectious viral content in tissues of animal models infected with NiV varies greatly. For NHP, the highest NiV titers observed have been 1 × 10^4^ PFU/mL [19,20], while for mice, the majority of research groups have encounter difficulties in finding an adequate mouse model susceptible to NiV [21]. Consequently, historically reported viral burdens in mice range from 1 × 10^2^ to 1 × 10^4^ PFU/mL in the most successful cases.

In this report, we describe a protocol for the inactivation validation of NiV-infected tissues by 10% Neutral Buffered Formalin (NBF), TRIzol, and TRIzol LS. Formalin is a solution of formaldehyde gas dissolved in water; it typically contains 37–40% formaldehyde by volume and may also contain a small amount of methanol as a stabilizer. Formalin is a strong and versatile disinfectant and preservative, and is routinely used to inactivate negative-sense RNA viruses. TRIzol and TRIzol LS are solutions composed of phenol and guanidinium isothiocyanate. These reagents can effectively disrupt cells and tissues, denature proteins, and separate the different biomolecules into distinct phases for subsequent purification, and they are used for various purposes, including the inactivation of negative-sense RNA viruses.

Our protocol adheres to established parameters previously validated for the inactivation of Nipah virus in tissues obtained from infected animals. Specifically, it aligns with prior reports by Edwards et al. and Widerspick et al. [16,17], who demonstrated effective viral inactivation using 10% NBF and TRIzol LS in high-viral-titer tissues collected from ferrets and mice, respectively, following infection with distinct Nipah virus strains.

## 2. Materials and Methods

### 2.1. Sample Preparation

For these studies, we used tissue samples from African Green Monkeys (*Chlorocebus aethiops*, [AGM]) infected with NiV. These were banked samples from previously conducted animal studies. The specimens employed included serum and brain tissue obtained from the left caudal, tight cranial, or left cranial regions. During the initial screening, we noted low or undetectable levels of infectious virus in the samples. Consequently, we opted to augment the samples with a high-titer virus stock for further analysis (1:1 *v*/*v* or g/v). For serum, 500 μL of serum was mixed with 500 μL of our NiV stock or 500 μL of DMEM (1:1 *v*/*v*). Approximately 1.3–4 g of brain tissue (corresponding to an estimated volume of 3.85 cm^3^ (height~1.5 cm; width~1.8 cm; depth~1.4 cm) was spiked by injecting undiluted virus stock using a 20 μL pipette loading tip that is comparable to a 29 G needle (length: 45 mm, tip outer diameter at narrow end: ~0.3–0.5 mm), dispensing 20 μL sequentially until reaching a 1:1 (g:mL) tissue-to-virus ratio. The pipette tip was inserted 1 cm into at least 10 areas of tissue to ensure delivery of the undiluted viral stock throughout the sample. It is important to note that, for tissues, virus spiking by microinjection does not recapitulate authentic infection because it bypasses receptor-mediated entry, lacks intracellular replication and host responses, produces non-physiological viral distribution, and predominantly represents extracellular virions, thereby failing to model the complex virus–host interactions present in infected tissues.

To assess whether microinjection artificially facilitated solution penetration into tissue, tissue samples derived from mice with an estimated weight of 0.5–0.7 g and volume of 0.6 cm^3^ (height~0.7–0.9 cm; width~0.8–1.0 cm; depth~0.7–0.9 cm) were spiked with 100 μL of a luminescent substrate immediately after its preparation (FFz, Promega, Madison, WI, USA, 0.44 μmol) either by superficial application or by injection. Tissue samples were subsequently embedded in 3 mL of a quenching solution (10× PBS, Gibco, Grand Island, NY, USA, supplemented with 100 μM ammonium chloride, MilliporeSigma, Burlington, MA, USA). Tissues were scanned in a Lago-X/IVIS Spectrum (Spectral Instruments Imaging, Tucson, AZ, USA), using parameters for luminescence for 1, 30, and 50 min. All samples tested showed the same levels of luminescence at early times post-exposure (Supplementary Figure S1, 1 min image). After 30 min post-exposure, we observe an increase signal on those samples that were microinjected with the substrate, in accordance with FFz intrinsic luminescence signal peaking within 2 to 6 min of preparation (Supplementary Figure S1, 30 min image). At 50 min post-exposure, tissues that received a superficial application of the substrate showed a more than 90% loss of detectable luminescent signal, indicating rapid and effective access of the quencher to surface-associated substrate. In contrast, at the same time point, tissues in which the substrate was delivered by injection retained luminescent signal at the same time point, showing inefficient access of quencher to injected regions (Supplementary Figure S1, 50 min image). These results demonstrate that injection does not inherently promote rapid solution penetration throughout tissue and instead can result in the retention of material within regions that are not immediately accessible to diffusible agents, underscoring the importance of tissue architecture and delivery method when interpreting inactivation kinetics.

### 2.2. Cells and Virus

African green monkey kidney cells (Vero E6, NR-596, BEI Resources, Manassas, VA, USA) were grown at 37 °C with 5% CO_2_ in normal growth media that consisted of Minimum Essential Media (Gibco, Grand Island, NY, USA) containing 2 mM l-glutamine (Gibco, Grand Island, NY, USA) and 1 mM sodium pyruvate (Gibco, Grand Island, NY, USA) with 10% heat-inactivated fetal calf serum (FCS, Gibco, Grand Island, NY, USA). Nipah virus, 200401066 Bangladesh was isolated from the throat of a male child in Bangladesh on 22 January 2004. The virus was passaged three times in Vero E6 cells prior to receipt at the NEIDL, generating a working viral stock with a titer of 6.9 × 10^6^ PFU/mL. All work with the infectious virus was performed in the Biosafety Level-4 laboratory at the National Emerging Infectious Diseases Laboratories (NEIDL), Boston, MA, USA.

### 2.3. Determination of Limit of Detection

**Figure 1:**
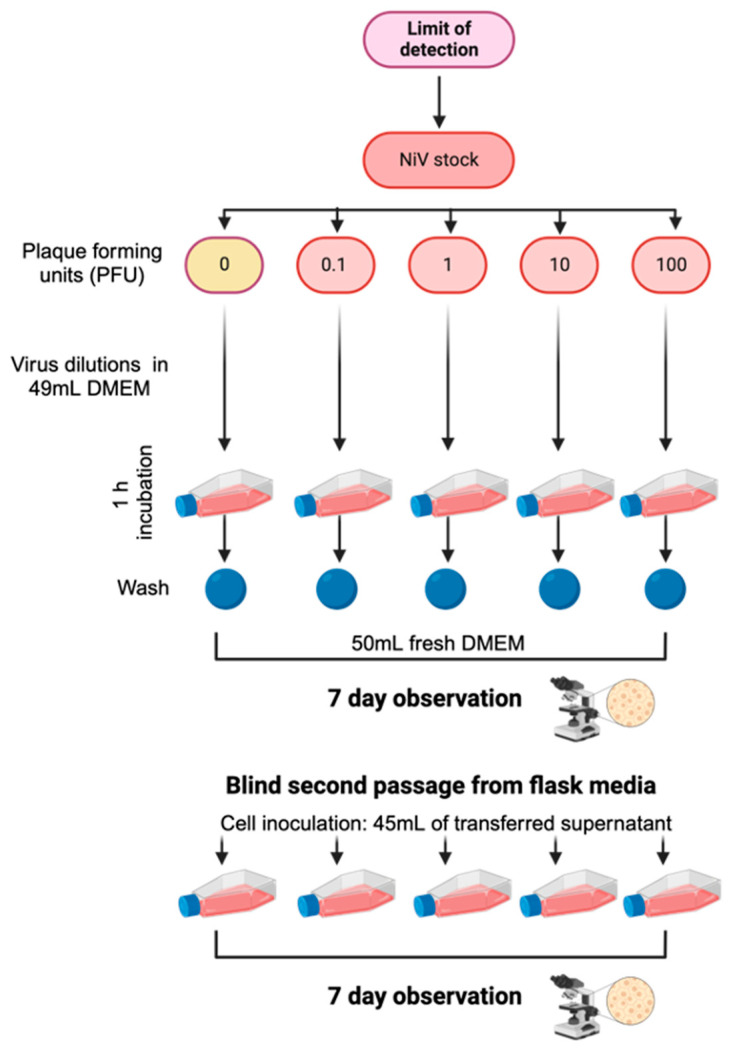
Experimental workflow to determine the limit of detection (LOD) of infectious NiV. Serial dilutions of NiV (target 0–100 PFU) were incubated on confluent cell monolayers for 1 h, washed, and overlaid with fresh DMEM. Cultures were monitored for cytopathic effect over 7 days, followed by the blind passage of flask supernatants onto fresh cells for an additional 7-day observation to assess infectious virus.

To evaluate the sensitivity of our experimental approach, we conducted a limit of detection (LOD) assay (Figure 1). Vero E6 cells were maintained in high-glucose DMEM (Gibco, Grand Island, NY, USA) supplemented with 1× GlutaMAX-I, 1 mM sodium pyruvate, 10% FBS, and 1× non-essential amino acids and were seeded onto 300 cm^2^ Tissue Culture Flasks at a density of 8.0 × 10^6^ cells per flask. The cells were incubated at 37 °C and 5% CO_2_ overnight. For this, 0, 0.1, 1, 10, or 100 plaque-forming units (PFU) of NiV were diluted in cell culture medium supplemented with 2% FBS for a final volume of 50 mL. The prepared solutions were used to inoculate the 300 cm^2^ flask containing Vero E6 cells seeded the prior day. After a 1 h incubation period, the inoculum was removed, washed three times with 50 mL of DPBS (Gibco, Grand Island, NY, USA), and fresh DMEM was added to the cells. Cells were incubated for 7 days at 37 °C and were monitored for cytopathic effects (CPE). Daily observations and microscope imaging were conducted throughout the week. After one week, the culture media from each plate were aspirated, and the entire volume was used to inoculate fresh Vero E6 cells seeded the prior day in a 300 cm^2^ flask. Observations and imaging of these cells continued until day 7 post-inoculation, as previously described [18].

### 2.4. Chemical Inactivation of NiV-Infected Samples

#### 2.4.1. 10% Neutral Buffered Formalin

**Figure 2:**
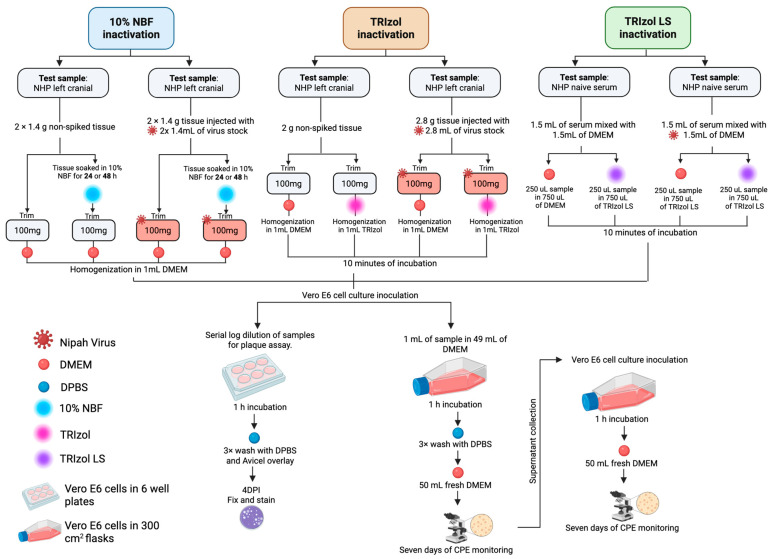
Schematic overview of chemical inactivation workflows for non-human primate (NHP) serum and tissue samples. Test tissues or serum were spiked with infectious NiV, then treated with 10% NBF, TRIzol, or TRIzol LS following standard inactivation protocols. After the indicated inactivation pipelines, samples were transferred to permissive cell cultures to assess residual infectivity by cytopathic effect or plaque formation as end points.

To evaluate the ability of 10% neutral buffered formalin (NBF) (LabChem, Zelienople, PA, USA) to inactivate infectious NiV, solid tissue samples containing a high content of NiV were treated with 10% NBF (Figure 2). Tissue specimens that were spiked with NiV or control were submerged in 10% NBF at a minimum ratio of 10 parts of fixative to 1 part tissue displaced volume. For this assay, individual tissues weighed less than 1.2 g and were inactivated in individual containers. After 24 or 48 h in 10% NBF at room temperature, the tissue was weighed and trimmed to <100 mg, 1 mL of media was added to the tube, and a Qiagen TissueLyser II (Qiagen, Hilden, Germany) was used for homogenization (two sets of 20–30 Hz for 2–3 min). The samples were then clarified by centrifuging for 1 min at 5000 RCF.

#### 2.4.2. TRIzol Reagent

To evaluate the ability of TRIzol reagent (Life Technologies, Carlsbad, CA, USA) to inactivate infectious NiV, solid tissue samples spiked with NiV were treated with TRIzol (Figure 2). As a negative control, we used tissues shown to lack detectable NiV and non-infected cells. Briefly, tissue was weighed and trimmed to <100 mg, 1 mL of TRIzol was added to the tube, and a Qiagen TissueLyser II (Qiagen, Hilden, Germany) was used for homogenization (two sets of 20–30 Hz for 2–3 min). Samples were then clarified by centrifuging for 3 min at 5000 RCF and then incubated at room temperature for 10 min for virus inactivation.

#### 2.4.3. TRIzol LS Reagent

To evaluate the ability of TRIzol LS reagent (Life Technologies, Carlsbad, CA, USA) to inactivate infectious NiV, liquid serum samples and clarified cell culture media containing NiV were treated using TRIzol LS (Figure 2). Serum derived from naïve animals and non-infected cell culture media were used as the negative control samples. Briefly, 250 μL of liquid was added to 750 μL TRIzol LS (ratio of 1:3, following the manufacturer’s instructions). Samples were mixed by vortexing for 3 s and incubated at room temperature for 10 min for virus inactivation.

### 2.5. Determination of Viral Titer by Plaque Assay

A plaque assay was used to quantify the infectious virus content of the samples. Briefly, Vero E6 cells were maintained in high-glucose DMEM (Gibco, Grand Island, NY, USA) supplemented with 1× GlutaMAX-I, 1 mM sodium pyruvate, 10% FBS (Gibco), and 1× non-essential amino acids (Gibco, Grand Island, NY, USA), and were seeded onto 6-well CellBIND plates (Corning, Corning, AR, USA) at a density of 8.0 × 10^5^ cells per well. The cells were incubated at 37 °C and 5% CO_2_ overnight. The media was removed from each well and replaced with 200 μL of 1 × 10^−2^ to 1 × 10^−6^ dilutions of the virus. One well containing only DMEM with 2% FBS was included as a control on each plate. Plates were incubated at 37 °C and 5% CO_2_ for 1 h with intermittent rocking. Cells were then overlaid with 2 mL of a 1:1 solution of 2.5% Avicel RC-591 (DuPont Nutrition and Health, Olathe, KS, USA) and 2× Temin’s Modified Eagle Medium (Gibco, Grand Island, NY, USA) without phenol red, supplemented with 10% FBS (Gibco, Grand Island, NY, USA), 2× antibiotic–antimycotic (Gibco, Grand Island, NY, USA), and 2× GlutaMAX-I (Gibco, Grand Island, NY, USA). The cells were incubated at 37 °C and 5% CO_2_ for 2 days. Plates were fixed in 10% NBF (ThermoFisher Scientific, Waltham, MA, USA), followed by staining with 0.2% Gentian Violet (Ricca Chemical, Arlington, TX, USA) in 10% NBF. The number of plaques per virus dilution was determined by eye and used to calculate the titer of the virus using the following formula:

$$\mathrm{Virus}\;\mathrm{titer}\;(\mathrm{PFU}/\mathrm{mL})=\frac{\mathrm{Number}\;\mathrm{of}\;\mathrm{plaques}}{\mathrm{Virus}\;\mathrm{dilution}\;\mathrm{in}\;\mathrm{well}\times \mathrm{Volume}\;\mathrm{plated}\;(\mathrm{mL})}$$

### 2.6. Analysis of Samples After Inactivation

To assess the presence of infectious NiV in treated samples in a highly sensitive manner, we performed two blind passages and monitored CPE, in accordance with our LOD approach described in Section 2.3 and Figure 2. Vero E6 cells maintained in high-glucose DMEM supplemented with 1× GlutaMAX-I (Gibco, Grand Island, NY, USA), 1 mM sodium pyruvate (Gibco, Grand Island, NY, USA), 10% FBS (Gibco, Grand Island, NY, USA), and 1× non-essential amino acids (Gibco, Grand Island, NY, USA) were seeded into 300 cm^2^ Tissue Culture Flasks at a density of 8.0 × 10^6^ cells per well. The cells were incubated at 37 °C and 5% CO_2_ overnight. Samples for each of the tested inactivation procedures (10% NBF, TRIzol, and TRIzol LS) were used to inoculate the flasks. Briefly, 1 mL of each sample was diluted in 49 mL of normal growth media containing 2% FBS (Gibco, Grand Island, NY, USA). The virus inoculum was added to the cells and allowed to incubate for 1 h at 37 °C. After this incubation period, the inoculum was removed, washed three times with 50 mL of DPBS (Gibco, Grand Island, NY, USA), and fresh DMEM was added to the cells. Cells were incubated for 7 days at 37 °C and were monitored for CPE. Daily observations and microscope imaging were conducted. After one week, all cell culture media from each flask were aspirated and used to inoculate naïve Vero E6 cells seeded the day prior in a 300 cm^2^ flask, with the entire volume from one plate deposited into one flask (45 mL). Observations and imaging of these cells continued until day 7 post-inoculation as previously described [18].

### 2.7. Assessing Inactivation Reagent Cytotoxicity and Impact on Virus Infection

**Figure 3:**
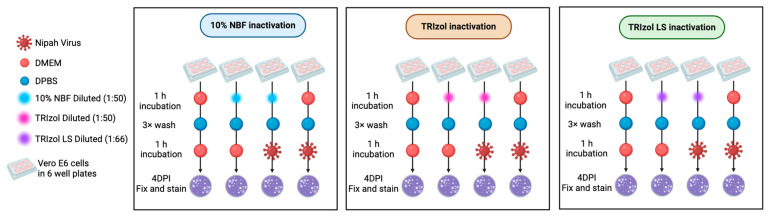
Experiment outline to assess the cytotoxicity of TRIzol, TRIzol LS, and 10% NBF treatments. Cell monolayers were exposed to each inactivation reagent for an hour, washed three times, and incubated in fresh DMEM before infection with 100 PFU of NiV. After a 1 h infection period, infectivity was measured by plaque assay.

To determine whether any of the treatments used in this study compromised the intrinsic ability of naïve cells to support NiV infection, we assessed viral infectivity following cell exposure to NBF 10%, TRIzol, and TRIzol LS (Figure 3). Vero E6 cells maintained in high-glucose DMEM supplemented with 1× GlutaMAX-I, 1 mM sodium pyruvate, 10% FBS, and 1× non-essential amino acids were seeded into 6-well plates at a density of 8.0 × 10^5^ cells per well. The cells were incubated at 37 °C and 5% CO_2_ overnight. Cell monolayers were incubated for 1 h at 37 °C with 200 μL of the various reagents used for NiV inactivation. As a negative control, we used 200 μL DMEM. Inactivation reagents were diluted in DMEM at the same ratio used in the chemical inactivation (Table 1). After 1 h incubation, inactivation reagents or DMEM overlay were removed, and cells were washed three times with 3 mL of DPBS following the procedure described in Section 2.6. Cells were then exposed to 200 μL of 1 × 10^−1^ to 1 × 10^−6^ dilutions of the NiV stock; infection and plaque assay proceeded as described in Section 2.5.

## 3. Results

### 3.1. Limit of Detection Determination

To determine the sensitivity for detection of infectious NiV particles across the different inactivation protocols, a limit-of-detection analysis was performed in parallel with each biological replicate of the inactivation experiments (Figure 1).

**Figure 4:**
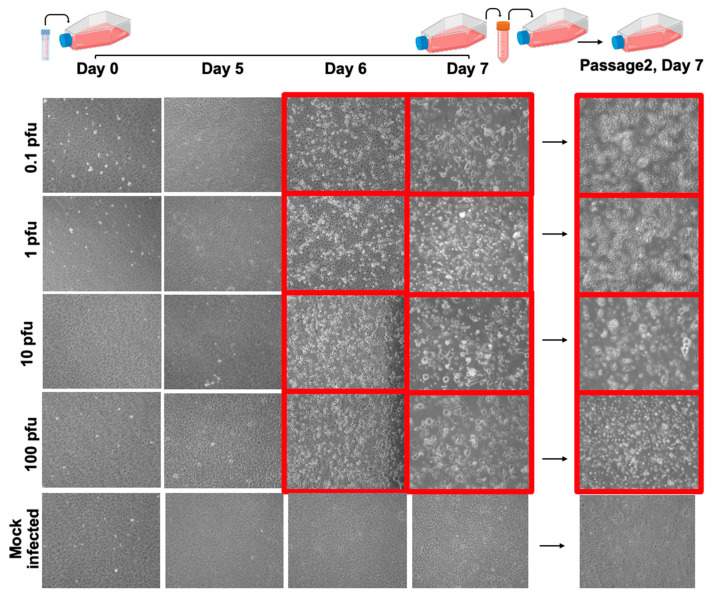
Representative 10× bright-field images from the NiV LOD assay. Cell monolayers were inoculated with different amounts of NiV (target 0.1–100 PFU) or mock control and imaged on Days 0, 5, 6 and 7. Red-boxed regions highlight the progression of cytopathic effect (CPE) at each PFU. Supernatants collected on Day 7 were blindly passed onto naïve cells, and representative 10× images from the blind passage (Day 7) are shown, confirming the presence or absence of infectious virus across tested conditions.

By day 6, cells exposed to 0.1, 1, 10, and 100 PFU of NiV displayed noticeable CPE (Figure 4, images highlighted in red frame). This intensified by day 7 post-infection, with a more pronounced CPE observed in cells infected with 10 and 100 PFU. In contrast, mock-infected flasks showed no CPE, but rather cell overgrowth (Figure 4, last row).

Supernatant derived from each tested flask was collected and blindly passaged to naïve Vero E6 cells. We observed complete whole monolayer disruption at day 7 in all the tested PFU; in contrast, cell monolayers exposed to supernatant derived from mock-infected cells showed no notable CPE. We can conclude that in this cell line, a blind passage generated from an infection of 0.1 PFU of NiV is sufficient to cause visible CPE 7 days post-infection, based on its comparison with blind passages derived from mock-infected cells.

### 3.2. Treatment with 10% NBF of NiV-Spiked Tissue Samples Inactivates NiV Infectious Particles

To test the ability of formalin to inactivate NiV, NHP tissue specimens (average weight of 1.4 g) were exposed to 10% NBF for 24 or 48 h, and then thoroughly washed to remove residual fixative. As described in Section 2.4.2, tissue specimens were then trimmed into 100 mg pieces and homogenized in 1 mL of DMEM. For each condition, there were at least 10 homogenized samples. Non-spiked and mock-treated controls were maintained in parallel (Figure 2). This experiment was repeated thrice for each condition, and we tested, in total, seven biological replicates of 100 mg.

**Figure 5:**
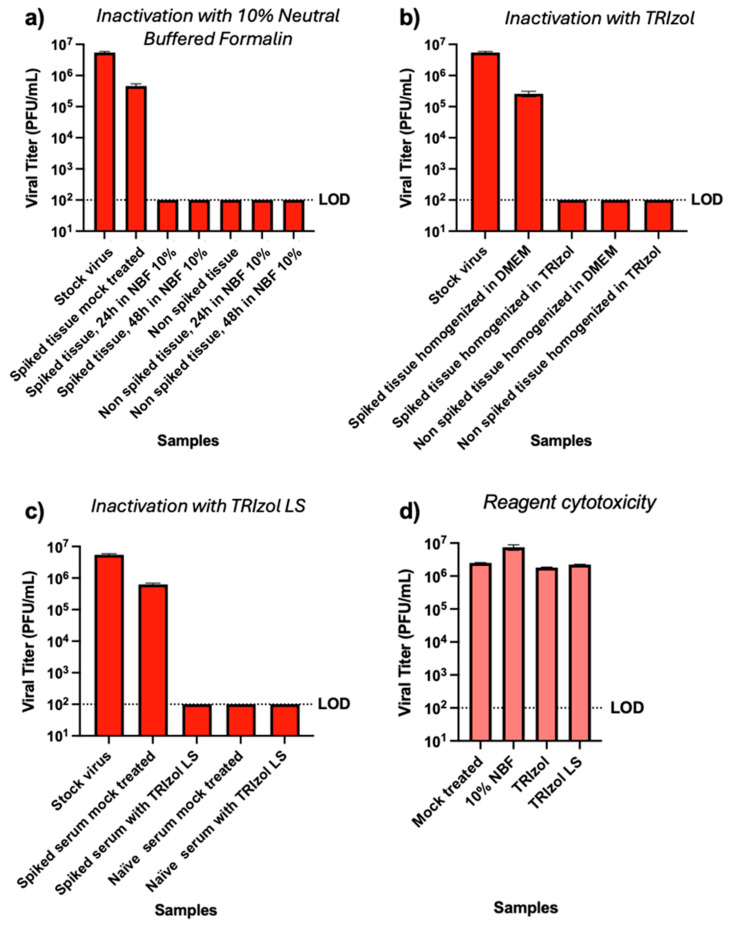
Infectious virus quantification from tissues treated with or without chemical inactivants. (**a**) Tissue homogenates, either untreated or soaked in 10% NBF; (**b**) tissue homogenized on DMEM or TRIzol; (**c**) serum samples treated with or without TRIzol LS; (**d**) inactivation reagent cytotoxicity evaluation. Samples were assessed for residual infectious NiV by plaque assay. Graphs show the average of three technical replicates per condition. A dotted line is shown to denote the limit of detection (LOD) for this assay.

We first quantified the viral titer of our prepared sample set by subjecting 100 μL of each sample to plaque assays. The stock virus yielded plaques after 4 days post-infection, with average titers of 5.48 × 10^6^ PFU/mL, while mock-treated spiked tissues yielded an average of 4.61 × 10^5^ PFU/g, confirming an efficient and homogeneous spiking throughout the tissue sample. In contrast, tissues submerged in 10% NBF for both 24 h and 48 h did not yield plaques after 4 days post-infection. Additionally, our set of negative controls did not yield any plaques at any dilution tested (Figure 5a, Table 2).

**Figure 6:**
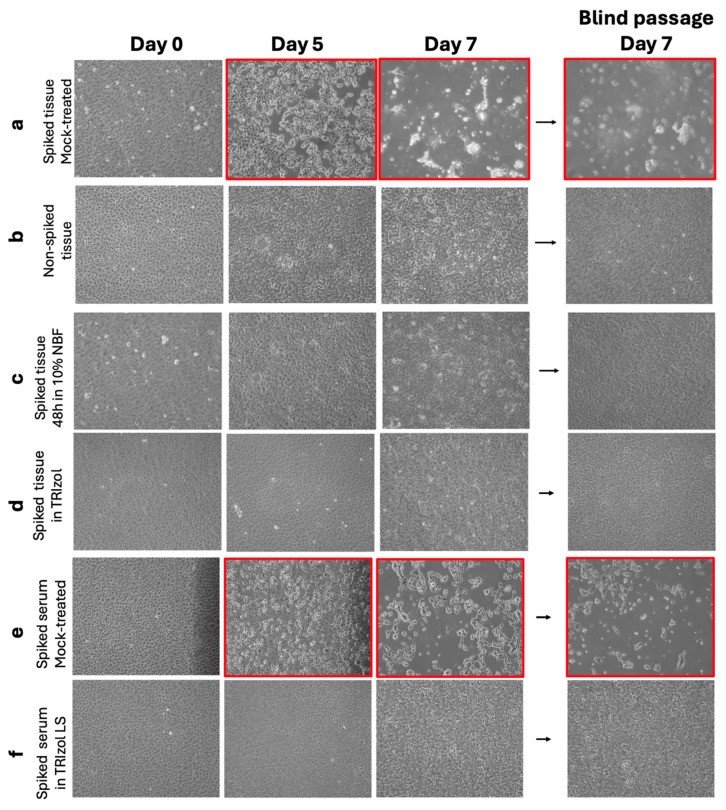
Representative 10× bright-field microscopy of samples assessing viral inactivation. (**a**) Spiked tissue, Mock-treated; (**b**) Non-spiked tissue; (**c**) Spiked tissue 48 h in 10% NBF; (**d**) Spiked tissue in TRIzol; (**e**) Spiked serum, Mock-treated; (**f**) Spiked serum in TRIzol LS. Cell monolayers were inoculated with the indicated samples and imaged on Days 0, 5 and 7. Red-boxed regions highlight the progression of cytopathic effect (CPE). Supernatants collected on Day 7 were blindly passed onto naïve cells, and representative 10× images from a blind passage (Day 7) are shown.

To assess the presence of infectious particles by a more sensitive assay, we monitored CPE generation by inoculating a larger cell culture format with 1 mL of our sample and performed a blind passage as described in Section 2.6 (Figure 2). Microscope imaging was conducted throughout the week. After 5 days of infection, we observed CPE on those cells incubated with the positive control: NiV-spiked tissue, mock-treated (Figure 6a,b, images highlighted in red frame).

By day 7, an extensive CPE was observed on our positive control samples, while the non-spiked tissues, included to evaluate cell appearance and CPE confirmation, did not exhibit CPE (Figure 6c).

In agreement with the observations of our LOD assays, the blind passage generated a strong CPE at 7 days post-inoculation in those flasks infected with mock-treated spiked tissue samples.

In contrast to the mock-treated spiked tissue samples, NiV-spiked tissues that were treated with 10% of NBF for 24 and 48 h did not show CPE after a blind passage (Figure 6). Together, these findings demonstrate that the treatment of NiV-spiked tissue samples with 10% NBF for either 24 or 48 h reliably and completely inactivates infectious NiV, as evidenced by the absence of detectable plaques or CPE across passages.

### 3.3. TRIzol Treatment of NiV-Spiked Tissue Samples Inactivates NiV Infectious Particles

To assess the efficacy of TRIzol-based chemical inactivation and downstream viral detection, right cranial lobe tissues from non-human primates (NHPs), spiked or not with NiV, were processed as depicted in Figure 2.

We first quantified the presence of NiV infectious particles in our sample set by plaque assay. The stock virus and positive control yielded plaques after 4 days post-infection, with average titers of 5.48 × 10^6^ PFU/mL and 2.6 × 10^5^ PFU/g, respectively. In contrast, tissues homogenized in TRIzol did not yield plaques after 4 days post-infection. Additionally, the negative controls did not yield plaques at any dilution tested (Figure 5b, Table 2).

We then proceeded to analyze further tissue samples after TRIzol activation. A 1 mL aliquot of these treatments was used to inoculate individual Vero E6 cells flasks and checked for signs of CPE (Figure 2).

Microscope imaging was conducted throughout the week, and after 5 days of infection, we were able to observe CPE on those cells incubated with the positive control: NiV-spiked tissue, mock-treated (Figure 6a,b, images highlighted in red frame). As expected, CPE increased through day 7. The negative control, non-spiked tissue, did not show signs of CPE in any of the observed days.

Consistent with our plaque assay observations, inoculation and blind passage of tissues after TRIzol treatment did not generate any detectable CPE (Figure 6d). Collectively, these results confirm that TRIzol treatment effectively and completely inactivates infectious NiV in spiked tissue samples, as demonstrated by the absence of plaques and the lack of CPE across primary infection and blind passages.

### 3.4. Serum Samples Spiked with NiV Were Inactivated After TRIzol LS Reagent Treatment

To determine the ability of TRIzol LS to inactivate NiV in serum, 250 μL of naïve or NiV-spiked serum was mixed with 750 μL of TRIzol LS (ratio of 1:3, following the manufacturer’s instructions) or DMEM, as described in Section 2.4.3 (Figure 2).

We then proceeded to quantify the presence of infectious particles in our set of samples. Plaque assays derived from the stock virus and positive control generated plaques after 4 days post-infection, with titers of 5.48 × 10^6^ and 6.30 × 10^5^ PFU/mL, respectively. Spiked serum exposed to TRIzol LS did not generate plaques after 4 days post-infection; similarly, the negative control, non-spiked serum treated with DMEM or TRIzol LS, did not generate plaques (Figure 5c, Table 2).

We then proceeded to analyze further samples after TRIzol LS inactivation by CPE monitoring and blind passages. A 1 mL aliquot of these treatments was used to inoculate Vero E6 cells flasks and checked for signs of CPE. Consistent with our plaque assay observations, inoculation and blind passage of untreated NiV-spiked serum generated high CPE at day 5 post-inoculation (Figure 6e, highlighted in red); when samples were treated with TRIzol LS, they yielded similar results as the negative controls used for this part of the study (Figure 6f).

### 3.5. Inactivation Reagents Used in This Study Do Not Have a Cytotoxic Effect in Cells and Do Not Affect Cells’ Susceptibility and Permissibility to NiV Infection

Inactivation reagents are often highly cytotoxic to cells in vitro when used at high concentrations. This cytotoxic effect could then impair cells’ basic functions and block viral replication even when infectious particles are still present in the sample used for inoculation. To evaluate the impact of the exposure of 10% NBF, TRIzol, and TRIzol LS in vitro, Vero E6 cell monolayers were exposed for an hour to the different inactivation reagents at the indicated dilutions in Table 1; those dilutions emulate our initial inactivation assay conditions described in Section 2.4 (Figure 3).

After exposure to different inactivation reagents or to DMEM, cells were processed and infected with serial dilutions of the NiV stock as described in Section 2.7. After 4 h post-infection, NiV titers were quantified by plaque assay (Figure 5d, Table 2).

We observed that the pre-treatment of cell monolayers with the inactivation reagents did not have an impact on NiV infectivity, and after plaque count and titer quantification, all the tested conditions displayed similar NiV titers (Figure 5d, Table 2). This experiment confirmed that with sufficient dilution, we were able to assess the presence of infectious viruses in infected samples, without the need for purifying columns.

## 4. Discussion

Various methods have been employed to assess the inactivation of viruses in samples with varying levels of sensitivity. Evaluating inactivation in the presence of toxic chemicals can be challenging because even small amounts of infectious virus could pose a public health risk and classify the sample as a Select Agent, especially when biosafety requirements are lowered, and strict inventory controls are no longer enforced. For example, less than 1 PFU of Ebola virus is lethal to nonhuman primates [19].

In this study, we utilized 10% NBF, TRIzol, and TRIzol LS reagents to fully inactivate NiV in infected tissues (Table 3), aligning with observations from the previously documented literature. The effectiveness of the viral inactivation process was validated through a plaque assay followed by two successive cell passages. We confirmed that incorporating two passages of inactivated material alongside samples containing minimal virus content (as little as 0.1 PFU) provided greater clarity in determining whether infectious material had been completely inactivated.

Our findings demonstrate that commonly used inactivation methods for Risk Group 4 negative-sense RNA viruses are both reliable and reproducible. Each institution and user is responsible for understanding and complying with FSAP regulations applicable to the specific agent being used, and the procedures described in this report will serve as valuable resources for other laboratories performing similar validation studies.

Our results confirm and extend to NHP-derived material the validation performed by Edwards et al. [16] with 4% paraformaldehyde for infected cell monolayers and 10% neutral-buffered formalin for infected ferret tissues, as effective inactivation agents for Hendra and Nipah viruses. Their study confirmed total loss of infectivity (≥8-log reduction) within short exposure periods (15 min using PFA 4%) for cell monolayers, after 24 h and 48 h, using 10% NBF for kidney and liver tissues, respectively; this correlates with the data shared in this study. Additionally, our results are in concordance with Widerspick et al. [17], where they extended this work by comparing nineteen physical and chemical conditions for Nipah virus in diverse matrices (cell cultures, supernatants, organ tissues). The study reinforced that multiple standard reagents (4% PFA, 1% SDS + heat, UV, acetone/methanol) can completely inactivate NiV, yet underlined that sample composition and viral load strongly influence efficacy, requiring matrix-specific confirmation and multi-passage validation.

In summary, the methods outlined in this study can be readily adopted by other laboratories to validate the inactivation of viruses within the Paramyxoviridae family across different matrices and chemical treatments, thereby supporting safe handling at lower biosafety containment levels.

## Figures and Tables

**Table 1:** Summary of treatment conditions and final dilutions applied to cell cultures. This table outlines the experimental treatment conditions, including the initial timepoint at which compounds were administered and the final dilution or concentration of each treatment to which the cells were exposed.

Section	Treatment	mL	DMEM mL	Dilution Factor
Section 2.4.1	10% NBF	1	49	0.02
Section 2.4.2	TRIzol	1	49	0.02
Section 2.4.3	TRIzol LS	0.75	49.25	0.015

**Table 2:** Summary of treatment conditions and results. Virus inactivation was assessed based on the presence or absence of cytopathic effect (CPE) in Vero cell monolayers following sample inoculation and subsequent blind passage. RT—room temperature; (−) no CPE observed; (+) CPE observed.

Sample	Treatment	Treatment Exposure Time	5DPI CPE	7DPI CPE	Blind Passage 7DPI CPE	Complete Inactivation
Mock Brain Tissue	None	NA	−	−	−	NA
Spiked Brain Tissue	None	NA	+	+	+	NA
Mock Brain Tissue	10% NBF	24 h, RT	−	−	−	NA
Mock Brain Tissue	10% NBF	24 h, RT	−	−	−	NA
Spiked Brain Tissue	10% NBF	48 h, RT	−	−	−	Yes
Spiked Brain Tissue	10% NBF	48 h, RT	−	−	−	Yes
Mock Brain Tissue	None	NA	−	−	−	NA
Spiked Brain Tissue	None	NA	+	+	+	NA
Mock Brain Tissue	TRIzol	15 min, RT	−	−	−	NA
Spiked Brain Tissue	TRIzol	15 min, RT	−	−	−	Yes
Mock Serum	None	NA	−	−	−	NA
Spiked Serum	None	NA	+	+	+	NA
Mock Serum	TRIzol LS	15 min, RT	−	−	−	NA
Spiked Serum	TRIzol LS	15 min, RT	−	−	−	Yes

**Table 3:** Inactivation methods used in this study to inactivate samples containing NiV.

Sample Nature	Inactivation Chemical	Amount of NiV
NHP tissues	10% Neutral Buffered Formalin	4.6 × 10^5^ PFU
NHP tissues	TRIzol reagent	4.9 × 10^5^ PFU
NHP serum	TRIzol LS reagent	6.3 × 10^5^ PFU

## Data Availability

Data are contained in the article.

## Supplementary Materials

The following supporting information can be downloaded at: https://www.mdpi.com/article/10.3390/v18010081/s1, Figure S1: Ex vivo scanning to assess tissue penetration using luminescent substrate spiking.
