# Supplementary material for: Validation of Chemical Inactivation Protocols for Henipavirus-Infected Tissue Samples
Source: Viruses. 2026 Jan 7;18(1):81. doi: 10.3390/v18010081 (PMC12846667; doi:10.3390/v18010081)
Supplement: Supplementary file 1 [file viruses-18-00081-s001.zip › viruses-4030489-supplementary.pdf]

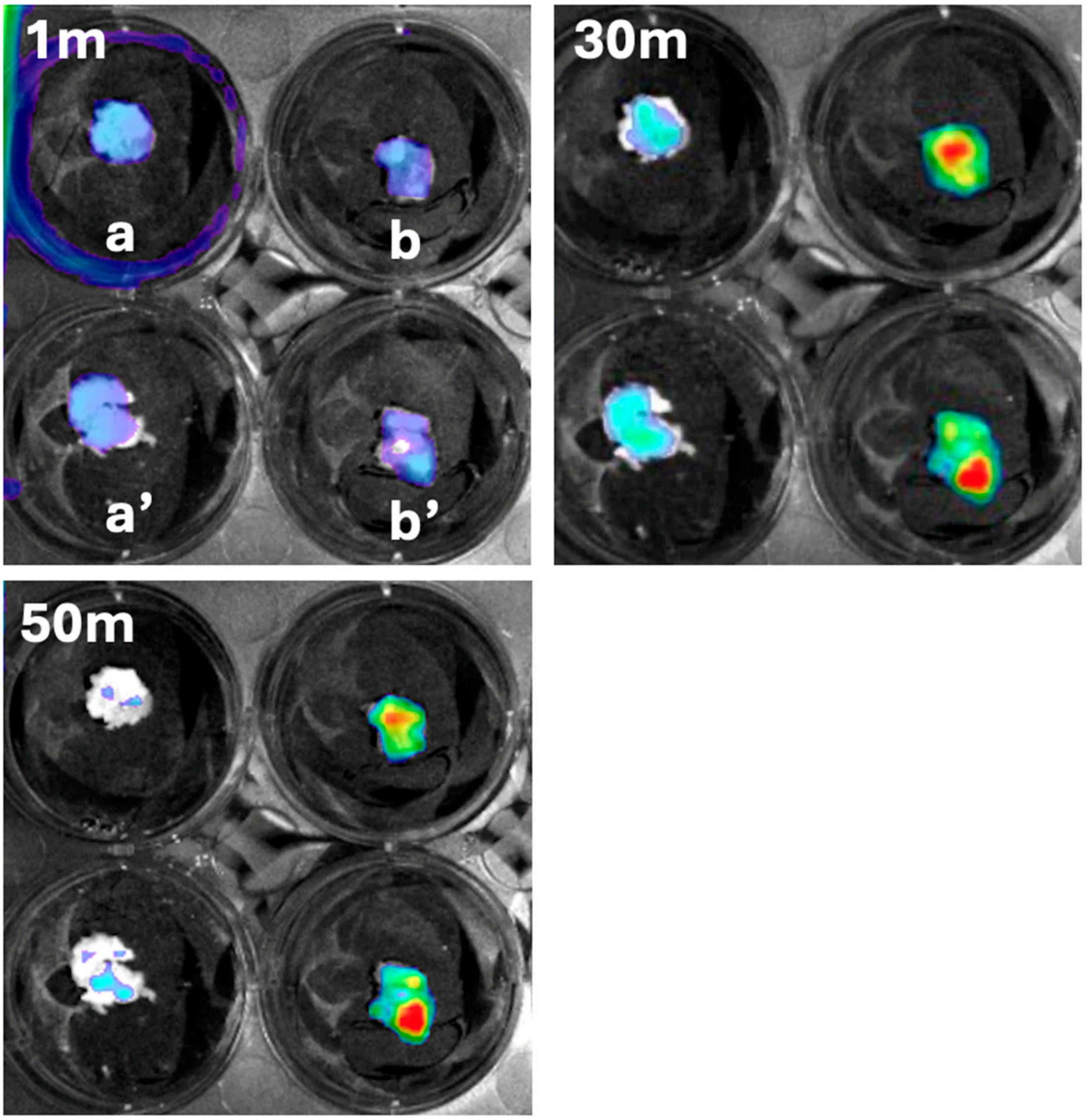

Supplementary Figure S1. *Ex vivo* scanning to assess tissue penetration using luminescent substrate spiking. *Ex vivo* tissue samples were spiked with 100μL of a luminescent substrate (FFz, 0.44 μmol) either by superficial (a, a’ panels) application or by microinjection (b, b’ panels.) Tissues were then exposed to a quenching solution (10X PBS supplemented with 100 μM ammonium chloride) for defined time intervals and imaged using an IVIS Spectrum system (open emission filter, FOV 22.4 × 22.4 cm, f/stop 1, binning M(8), exposure 1–20 s). Residual luminescent signal was used as a proxy for solution penetration into tissue and to compare the impact of delivery method on accessibility over time. Scanning of 1 minute, 30 minutes, 45 minutes and 60 minutes post exposure times are showing.
